# [Corrigendum] Furin promotes epithelial-mesenchymal transition in pancreatic cancer cells via Hippo-YAP pathway

**DOI:** 10.3892/ijo.2025.5797

**Published:** 2025-08-26

**Authors:** Youli Zhang, Meng Zhou, Hong Wei, Hailang Zhou, Junbo He, Ying Lu, Dawei Wang, Baoding Chen, Jian Zeng, Wanxin Peng, Fengyi Du, Aihua Gong, Min Xu

Int J Oncol 50: 1352-1362, 2017; DOI: 10.3892/ijo.2017.3896

Following the publication of the above article, the authors drew to the Editor's attention that the image in Fig. 3A on p. 1356 for the 'Migration/BxPC3/sh-EGFP' experiment was mistakenly presented. This error arose as a consequence of a mistake that was made during the preparation of the final images. Furthermore, upon performing an independent analysis of the data in this paper in the Editorial Office, it came to light that, for the colony-formation assay experiments shown in Fig. 2F on p. 1355, the image selected for the 'PaTu8988/Flag-Furin' experiment had already appeared in a different context in another paper published by the same authors, also in the journal *International Journal of Oncology*.

After having examined their original data, the authors realize that this second figure in the paper had also been inadvertently assembled incorrectly. The revised versions of Fig. 2 (now showing the data correctly for the for the 'PaTu8988/ Flag-Furin' experiment) and Fig. 3 (showing the correct data for the 'Migration/BxPC3/sh-EGFP' experiment) are shown on the next two pages. Note that the errors made during the compilation of these figures did not affect the overall results and conclusions reported in the paper. The authors are grateful to the Editor of *International Journal of Oncology* for granting them the opportunity to publish this corrigendum, and all the authors agree with its publication; furthermore, they apologize to the readership of the journal for any inconvenience caused.

## Figures and Tables

**Figure 2:**
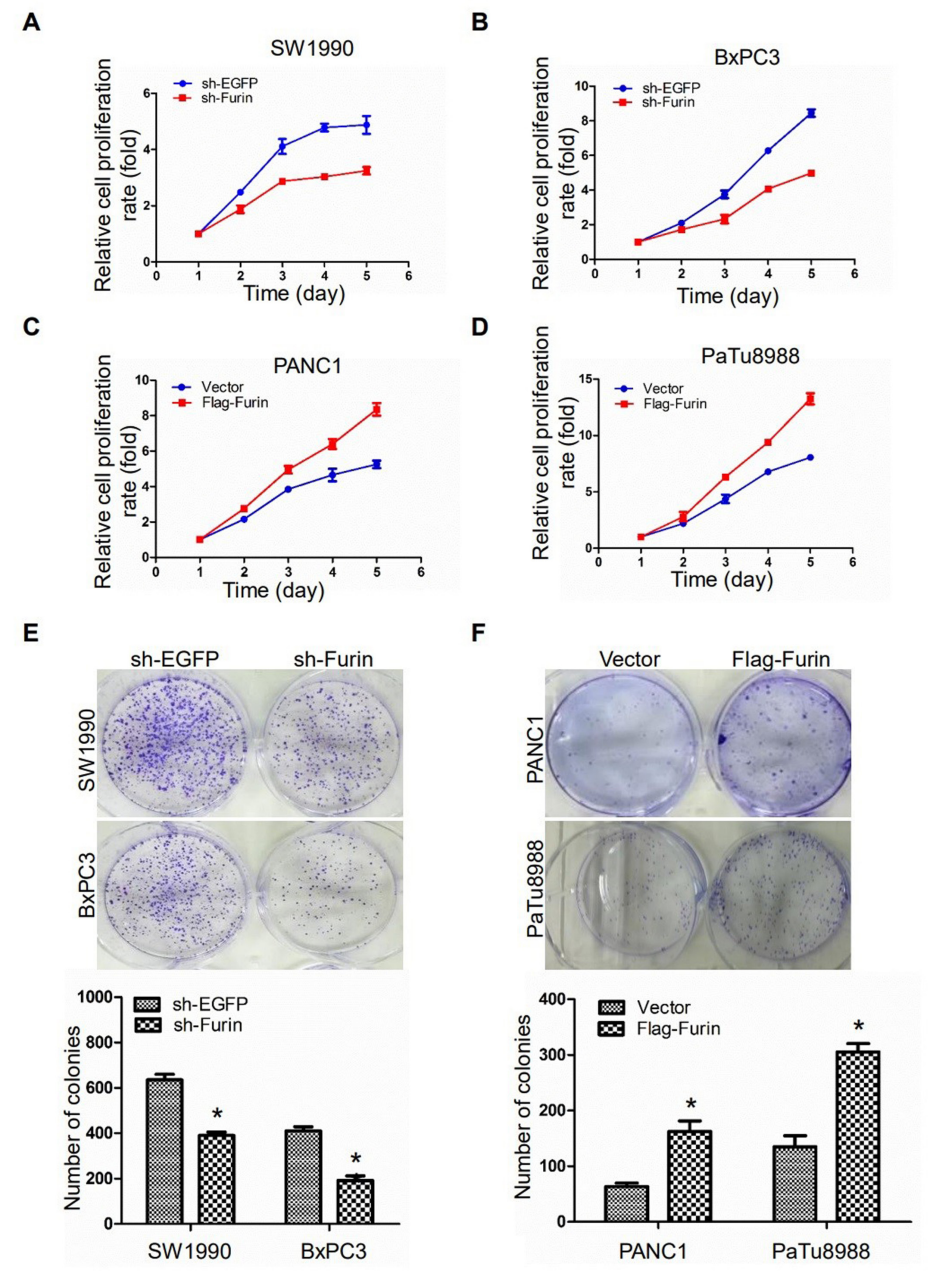
Furin promotes cell proliferation in pancreatic cancer cells. (A and B) CCK-8 assay showed that Furin knockdown inhibited BxPC3 and SW1990 cell growth rate, the data are presented as the mean ± SD; (n=3). (C and D) CCK-8 assay showed that Furin ovexpression promoted cell proliferation rate in PANC1 and PaTu8988 cells, the data are presented as the mean ± SD (n=3). (E) Colony forming assay showed that Furin knockdown inhibited BxPC3 and SW1990 cell anchorage-independent growth, the data are presented as the mean ± SD (Student's t-test: ^*^P<0.05 vs. sh-EGFP). (F) Colony forming assay showed that Furin ovexpression promoted PANC1 and PaTu8988 cell anchorage-independent growth, the data are presented as the mean ± SD (Student's t-test: ^*^P<0.05 vs. vector). The data were obtained from at least three independent experiments.

**Figure 3:**
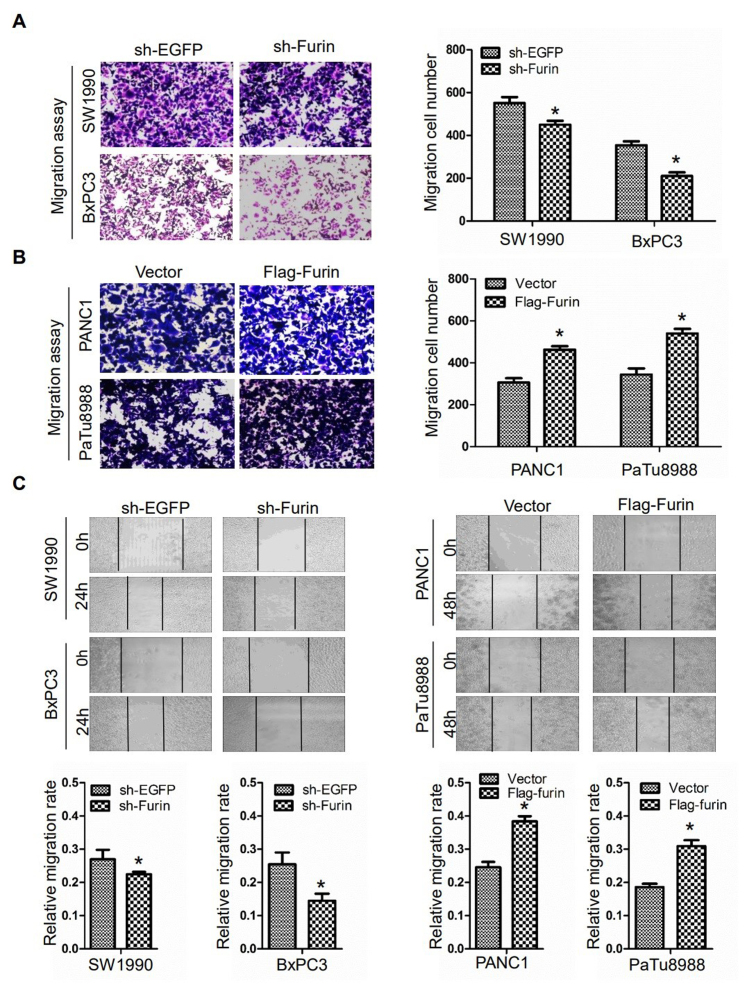
Furin promotes cell migration in pancreatic cancer cells. The migration changes was measured by Transwell migration assay (A and B) and wound healing assay (C), after transfected with sh-EGFP or sh-Furin plasmid in BxPC3 and SW1990 cells and vector or Flag-Furin plasmid in PANC1 and PaTu8988 cells, the data are presented as the mean ± SD (Student's t-test: ^*^P<0.05 vs. sh-EGFP, ^*^P<0.05 vs. vector). The data were obtained from at least three independent experiments.

